# Determinants of persistent post-COVID-19 symptoms: value of a novel COVID-19 symptom score

**DOI:** 10.1186/s43168-020-00049-4

**Published:** 2021-02-05

**Authors:** Islam Galal, Aliae A. R. Mohamed Hussein, Mariam T. Amin, Mahmoud M. Saad, Hossam Eldeen E. Zayan, Mustafa Z. Abdelsayed, Mohamed M. Moustafa, Abdel Rahman Ezzat, Radwa E. D. Helmy, Howida K. Abd_Elaal, Nasrallah A. Al Massry, Mohamed A. Soliman, Asmaa M. Ismail, Karima M. S. Kholief, Enas Fathy, Maiada K. Hashem

**Affiliations:** 1 Aswan Faculty of Medicine, Aswan University, Aswan, Egypt; 2 Chest Department, Assiut University Hospitals, Assiut, 71515 Egypt; 3 Assiut Faculty of Medicine, Assiut University, Assiut, Egypt; 4 Gastroenterology and infectious disease Department, Assiut University Hospitals, Assiut, Egypt; 5 Assiut University Hospitals, Assiut, Egypt; 6 Medical Pharmacy, Assiut University, Assiut, Egypt; 7 Assiut University, Assiut, Egypt; 8 Al Quds University, Alazhar University, Gaza Branch, Gaza, Palestine; 9 Department of Cardiovascular Medicine, Faculty of Medicine, Aswan University, Aswan, Egypt; 10 Chest Department, Assiut Faculty of Medicine, Assiut University Hospitals, Assiut, Egypt

**Keywords:** Post-COVID-19 symptoms, Symptom score, COVID-19 symptom score, Comorbidities, Persistent symptoms

## Abstract

**Background:**

Being a newly emerging disease, little is known about its long-lasting post-COVID-19 consequences. The aim of this work is to assess the frequency, patterns, and determinants of persistent post-COVID-19 symptoms and to evaluate the value of a proposed novel COVID-19 symptom score. Patients with confirmed COVID-19 in a hospital-based registry were included in a cross-sectional study (the hospitals including Assiut University Hospital, Assiut Chest Hospital, Aswan University Hospital, and Aswan Specialized Hospital). The patient demographics, comorbid disorders, the mean duration since the onset of the symptoms, history of hospital or ICU admittance, and the treatment taken during the acute state, as well as symptom score before and after convalescence, were recorded.

**Results:**

The most frequent constitutional and neurological symptoms were myalgia (60.0%), arthralgia (57.2%), restriction of daily activities (57.0%), and sleeping troubles (50.9%), followed by anorexia (42.6%), chest pain (32.6%), gastritis (32.3%), cough (29.3%), and dyspnea (29.1%). The mean total score of acute stage symptoms was 31.0 ± 16.3 while post-COVID 19 symptom score was 13.1 ± 12.6 (*P* < 0.001). The main determinants of the persistent post-COVID-19 symptoms were the need for oxygen therapy (*P* < 0.001), pre-existing hypertension (*P* = 0.039), chronic pulmonary disorders (*P* = 0.012), and any chronic comorbidity (*P* = 0.004). There was a correlation between the symptom score during the acute attack and post-COVID-19 stage (*P* < 0.001, *r* = 0.67). The acute phase score had 83.5% sensitivity and 73.3% specificity for the cutoff point > 18 to predict occurrence of post-COVID-19 symptoms.

**Conclusions:**

COVID-19 can present with a diverse spectrum of long-term post-COVID-19 symptoms. Increased acute phase symptom severity and COVID-19 symptom score > 18 together with the presence of any comorbid diseases increase the risk for persistent post-COVID-19 manifestations and severity.

## Background

Being a newly emerging disease a little known about long-lasting post-COVID-19 infection consequences. However, in the coming days, great stress will progressively comprise post-acute carefulness of those recovered cases from COVID-19. It is expected that COVID-19 may have a principal effect on the physical, mental, cognitive, and public health state [1]. Moreover, numerous serious COVID-19 infections necessitate intensive care unit (ICU) management and may lead to persistent post-convalescence consequences comprising respiratory, somatic, mental, and emotional abnormalities which are known as post-intensive care syndrome [2–4]. Recent studies illustrated that in patients who had convalesced from COVID-19, about 50–87% experienced persistence of at least one symptom, predominantly lethargy and shortness of breath that may necessitate some form of constant carefulness to recover their long-lasting consequences [5, 6]. High blood pressure, obesity, and mental health conditions are proposed risk factors for persistent post-COVID symptoms; however, surprisingly, even young adults and children without underlying chronic medical conditions and those with mild COVID-19 illness reported that they had not returned to their usual state of health several weeks after convalescence [7]. So, further researches are needed to understand the risk factors and pathophysiology of these persistent post-COVID-19 symptoms.

The aim of this work is to assess the frequency, patterns, and determinants of persistent post-COVID-19 symptoms and to evaluate the value of a proposed novel COVID-19 symptom score.

## Methods

The present study was a cross-sectional study performed from 18th of July to 31st of August 2020. Patients were included if they had confirmed COVID-19 in a hospital-based registry (positive or indeterminate COVID-19 PCR test, or presumed presence of COVID-19 based on clinical and radiological criteria). They were interviewed in the follow-up clinics (the outpatient clinics in the following hospitals including Assiut University Hospital, Assiut Chest Hospital, Aswan University Hospital, and Aswan Specialized Hospital) and filled paper follow-up forms in Arabic language (which is a special form that we formulated for our study). Medical students, residents, and volunteers evaluated patient’s symptoms and revised the submitted forms for missing data.

*Sample size* was calculated using Epi info statistical package version 7. Based on the number of cases in Egypt on 18th July, 2020 (86,474), the following parameters for cross-sectional study were expected cases with 0.50, with acceptable margin of error 0.05, design effect 1, 95% confidence level. The required sample size was 384 patients. It was raised to 425 after considering 10% as a dropout.

The following data were collected:

- The patient demographics including age, gender, body mass index, smoking status, history of comorbid disorders, the mean duration since the onset of the symptoms, history of hospital or ICU admittance, and treatment taken during the acute attack were recorded
- Symptoms during the acute attack of COVID-19.
- Symptoms after the convalescence from the acute attack of COVID-19 including general, upper, lower respiratory tract, neurological, cutaneous complaints, and symptoms suggesting other systems of the body affected.

### Symptom score

Two scores were used. Acute stage symptoms include 27 symptoms and post-COVID symptoms include 29 symptoms (comprising newly diagnosed DM and skin rash in addition to the acute stage symptoms). A 4-point Likert scale was used for each symptom reported as absent, mild, moderate, or severe (the score were designed and validated by 2 public health and statistical specialists in Asyut University). For acute stage and post-COVID symptoms, the range of the overall score is 0–81 and 0–87, respectively (the higher the number, the further symptom severity).

The study was approved by the ethical committee of Aswan Faculty of Medicine, Egypt (IRB number: aswu/469/7/2020) and registered in ClinicalTrial.gov: NCT04479293.

### Statistical analysis

Statistical analyses were performed using IBM SPSS Statistics version 20 (SPSS Inc., Chicago, IL, USA). Categorical data were presented as numbers and percentages, while continuous data were reported as means ± SD and/or median (min-max) and tested for normality using the Shapiro-Wilkes test. As symptom score was not normally distributed the Mann-Whitney and Kruskal-Wallis tests were used to compare score according to different variables. Also, Spearman’s correlation was used to find the correlation between symptom score in acute and post-COVID-19 stages. To detect the sensitivity and accuracy of acute stage symptom score in the prediction of persistent post-COVID symptoms, ROC curve analysis was performed. In all statistical tests, *p* value < 0.05 was considered statistically significant.

## Results

**Fig. 1:**
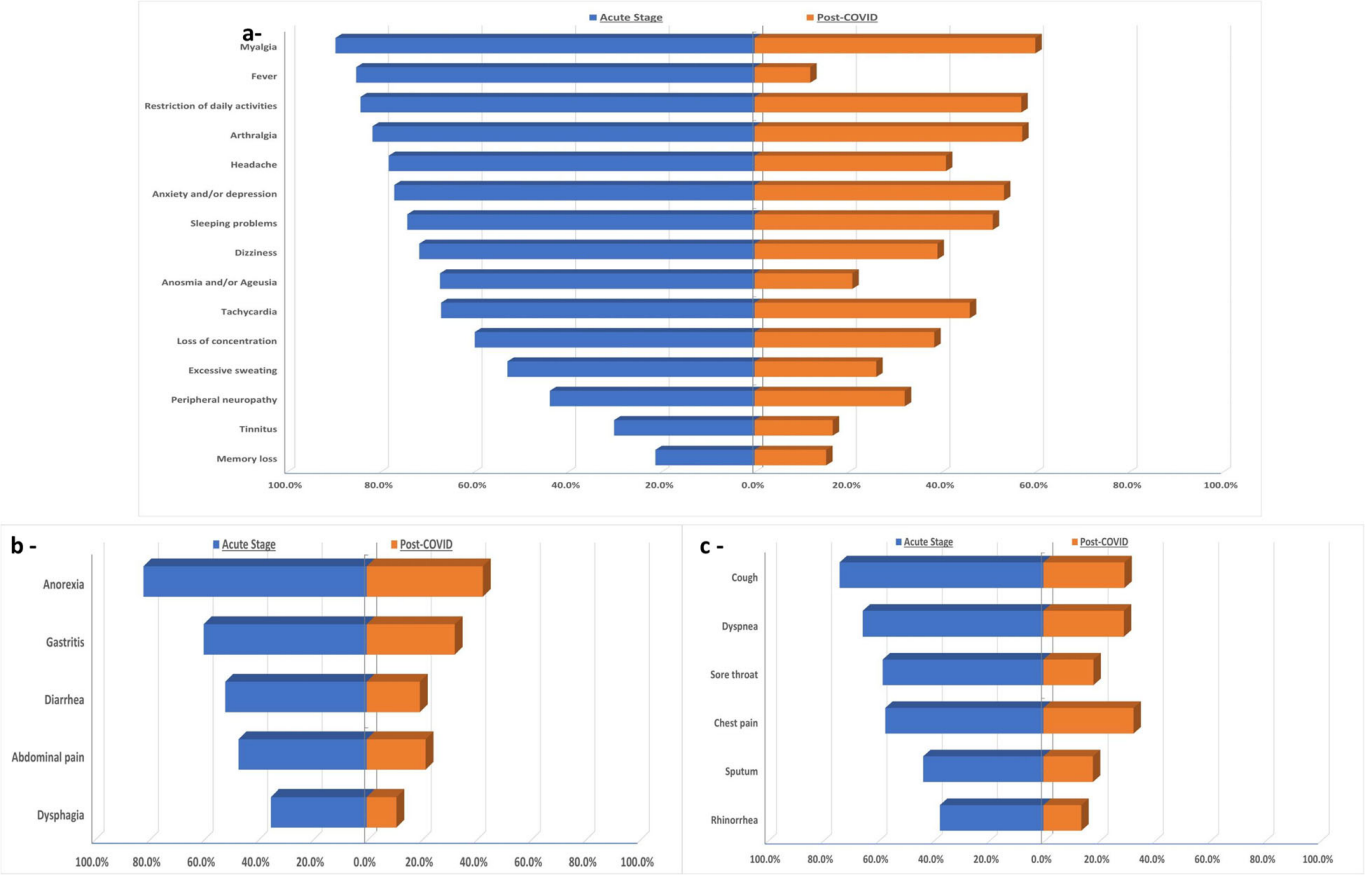
Reported symptoms in acute and post- COVID stages: **a** general symptoms, **b** GIT symptoms, **c** respiratory symptoms

The study involved 430 participants. They were 156 males and 274 females, their mean age was 37.4 ± 12.6 years, and the range was 12–74 years. The most common presenting symptoms during the acute attack were myalgia (89.5%), fever (85.1%), and restriction of daily activities (84.9%), while the least frequent complaint was memory loss (21.2%). Among them, 370 (86%) reported persistent post-COVID-19 symptoms*.* The most frequent constitutional and neurological symptoms were myalgia (60.0%), arthralgia (57.2%), restriction of daily activities (57.0%), sleeping troubles (50.9%), and nervousness and hopelessness (53.3%), while the most common respiratory and GIT symptoms were anorexia (42.6%), chest pain (32.6%), gastritis (32.3%), cough (29.3%), and dyspnea (29.1%), while the least frequent symptom was newly diagnosed DM (8.8%) (Fig. 1a–c). The mean total score of acute stage symptoms was 31.0 ± 16.3 while post-COVID 19 symptom score was 13.1 ± 12.6 (*P* < 0.001).

The mean duration of acute phase in the included group was 9 ± 3.1 days; the mean convalescence period was 7 ± 2.4 days, while the mean duration of persistent post-COVID-19 symptoms after recovery was 176 ± 35.1 days. The main essential determinant of the persistent post-COVID-19 symptoms and symptom score among the included patients was the need for oxygen therapy (*P* < 0.001) as shown in Table 1.

**Table 1:** Determinants of persistent post-COVID-19 symptoms and symptom score in the included patients (*n* = 430)

	***N*** (%)	Mean score	***P*** value*
**Age (years)**			
**< 25**	58 (13.5%)	12.9 ± 13.5	0.393
**25–40**	227 (52.8%)	12.6 ± 12.3	
**> 40**	145 (33.7%)	14.0 ± 12.7	
**Gender**			
**Male**	156 (36.3%)	13.2 ± 12.5	0.998
**Female**	274 (63.7%)	13.1 ± 12.6	
**BMI**			
**Underweight**	147 (34.2%)	11.9 ± 13.6	0.107
**Normal**	120 (27.9%)	13.6 ± 11.4	
**Overweight**	52 (12.1%)	14.4 ± 15.2	
**Obese**	66 (15.3%)	14.5 ± 12.6	
**Smoking**			
**Nonsmoker**	371 (86.3%)	12.9 ± 12.8	0.138
**Current smoker**	26 (6%)	13.3 ± 10.8	
**Ex-smoker**	33 (7.7%)	16.1 ± 11.8	
**Hospital admission during illness**			
**Yes**	103 (24%)	14.0 ± 12.4	0.216
**No**	327 (76%)	12.8 ± 12.7	
**Need of oxygen therapy**			
**Yes**	72 (16.7%)	17.4 ± 12.5	< 0.001^^^
**No**	358 (83.3%)	12.3 ± 12.4	
**ICU admission**			
**Yes**	20 (4.7%)	17.3 ± 12.3	0.066
**No**	410 (95.3%)	12.9 ± 12.6	

*Mann-Whitney and Kruskal-Wallis tests were uses

^**^**^Significant *p* value

**Fig. 2:**
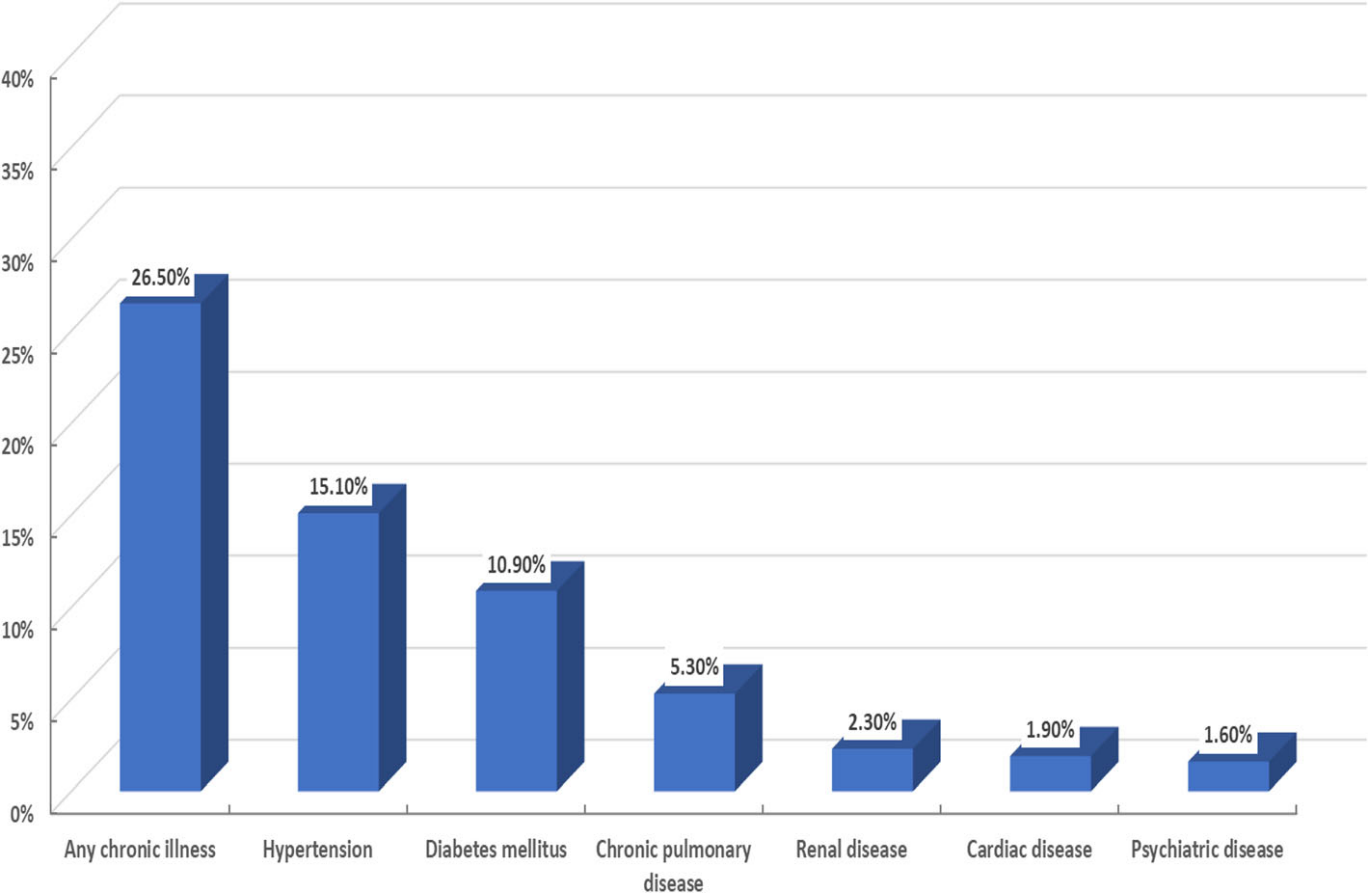
Associated comorbidities in post-COVID-19 patients included in the study (*n* = 430)

There were 26.5% patients who reported that they have a chronic illness, and the distribution of those conditions is illustrated in Fig. 2. The most frequent pre-existing comorbidities allied with the persistent post-COVID-19 symptoms and symptom score among the study population were hypertension (*P* = 0.039) followed by chronic pulmonary disorders (*P* = 0.012), and lastly, the presence of any chronic disorder (*P* = 0.004), as shown in Table 2. There was no difference in post-COVID-19 symptoms and symptom score despite the difference in the received treatment (supportive treatment, hydroxychloroquine, azithromycin, or corticosteroids).

**Table 2:** Post-COVID-19 symptom score in patients according to comorbidities (*n* = 430)

	Yes	No	***P*** value*
**Diabetes mellitus**	13.4 ± 11.8	13.1 ± 12.7	0.730
**Hypertension**	15.4 ± 12.9	12.7 ± 12.5	0.039^^^
**Cardiac disease**	13.8 ± 4.9	13.1 ± 12.7	0.276
**Chronic pulmonary disease**	20.5 ± 15.6	12.7 ± 12.3	0.012^^^
**Renal disease**	16.5 ± 11.9	13.1 ± 12.6	0.278
**Psychiatric disease**	22.0 ± 14.2	12.9 ± 12.5	0.068
***Any chronic illness***	15.8 ± 13.6	12.2 ± 12.1	0.004^^^

“Any chronic illness” means the presence of any comorbid chronic disease including DM, HTN, cardiac, chronic pulmonary, and psychiatric diseases

*Mann-Whitney test were uses

^**^**^significant *p* value

**Fig. 3:**
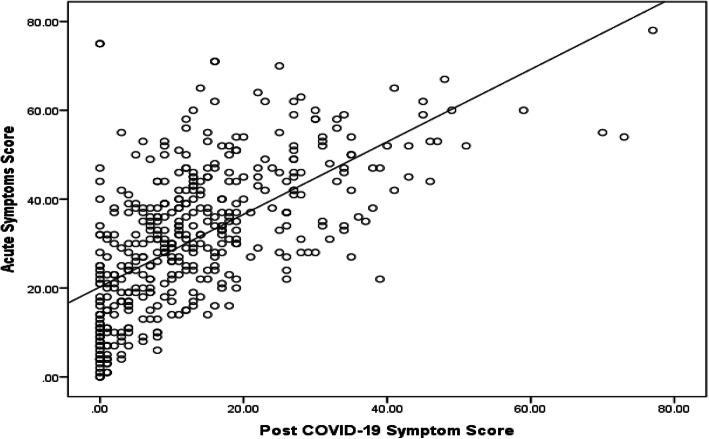
Correlation between symptom score during acute and post-COVID-19 stage

There was a strong positive correlation between the symptom score during the acute attack and post-COVID-19 stage (*P* < 0.001, *r* = 0.67) as illustrated in Fig. 3.

**Fig. 4:**
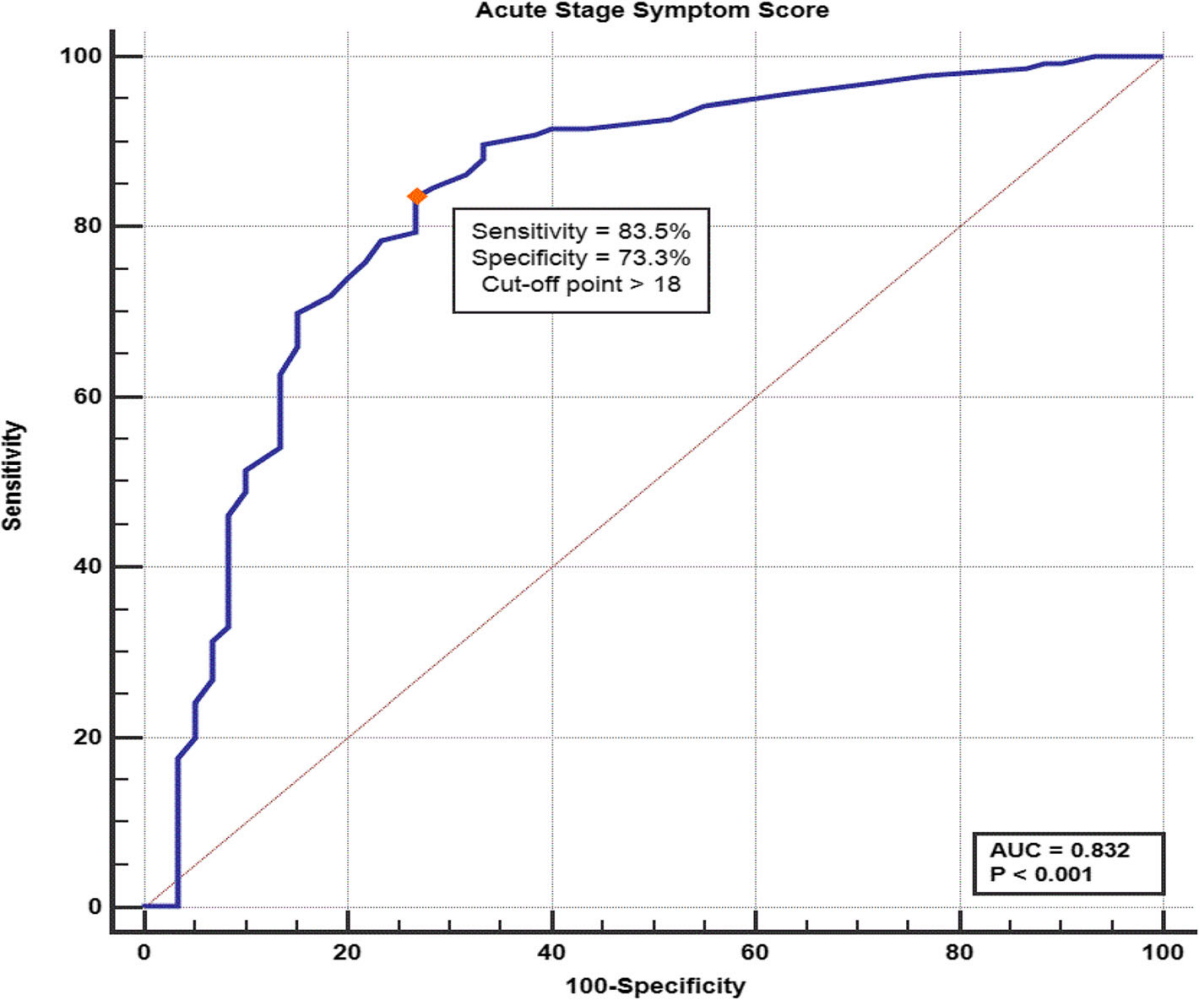
ROC curve for the acute stage symptom score as a predictor of persistent post-COVID symptoms

The acute phase score had 83.5% sensitivity and 73.3% specificity for the cutoff point > 18 to predict the occurrence of post-COVID symptoms (Fig. 4).

## Discussion

COVID-19 is a novel illness with limited information on the post- COVID-19 symptoms. The aim of the current study was to identify the essential determinates of the different patterns of these symptoms. Most of the studied population had been still complaining of several persistent post-COVID-19 symptoms. The most frequent constitutional and neurological symptoms were myalgia (60.0%), arthralgia (57.2%), restriction of daily activities (57.0%), sleeping troubles (50.9%), and nervousness and hopelessness (53.3%), while the most common respiratory and GIT symptoms were anorexia (42.6%), chest pain (32.6%), gastritis (32.3%), cough (29.3%), and dyspnea (29.1%). The mean total score of these post-COVID-19 symptoms was 13.1 ± 12.6. The main essential determinants of the persistent post-COVID-19 symptoms and symptom score among the included patients were previous seasonal influenza vaccination (*P* = 0.003) and the need for oxygen therapy (*P* < 0.001)***.*** Moreover, the most frequent pre-existing comorbidities allied with the persistent post-COVID-19 symptoms and symptom score among the study population were hypertension (*P* = 0.039), followed by chronic pulmonary disorders (*P* = 0.012), and lastly, the presence of any chronic disorder (*P* = 0.004). There was a strong positive correlation between the symptom score during the acute attack and post-COVID-19 stage (*P* < 0.001, *r* = 0.67). Furthermore, the acute phase score had 83.5% sensitivity and 73.3% specificity for the cutoff point > 18 to predict the occurrence of post-COVID symptoms.

By reviewing available literature, about 87% of those who recovered from COVID-19 infection were still suffering at least one symptom 1 to 2 months after disease onset. A wide spectrum of symptoms was reported including lethargy, breathing difficulty, cough, palpitations/tachycardia, chest pain, sleeping troubles, headache, joint ache, and deterioration of physical and mental well-being [5, 8–12].

On the other hand, an initial report of COVID-19 post-discharge complaints in China summarized that 86.2% of cases were asymptomatic while only 9.1 had cough and 1.5% had breathing difficulty that did not affected neither daily activities nor sleep, while dizziness, headache, and lethargy were not reported by those patients at all [13].

The duration of symptom resolution in included COVID-19 cases appears to be longer than that seen in community-acquired pneumonia caused by bacterial pathogens. Previous studies in patients with community-acquired pneumonia found that 97% of their symptoms recovered by an average of 10 days, while dyspnea resolved after an average of 2 weeks from the onset of the symptom, and lethargy after 3 weeks [14, 15].

It is not yet clearly recognized why some patients have persistent recovery. Long-lasting viremia owing to vague or weak or immunological reaction [16], relapse or re-contamination [17], inflammatory and other immunological responses [18, 19], de-conditioning [20], and psychological elements such as post-trauma stress syndrome may all added [21, 22]. Moreover, severe COVID-19 infections necessitate management in ICU and may lead to persistent post-recovery sequelae including respiratory, physical, mental, and psychological disorders [2, 3]. These complaints are stated to as post-intensive care syndrome (PICS); these sequelae can have persistent implications on the life quality [4]. Patients suffering PICS commonly report higher prevalence of mental and physical disorders, which may often be long-standing [23]. PICS can also cause disability and reasonable pain [24]. According to Murray et al., about half percent of hospitalized patients for COVID-19 will necessitate constant care to ameliorate their long-standing consequences [6].

Post-recovery symptoms may also be predicted from the preceding coronavirus epidemics of severe acute respiratory syndrome. SARS survivors still had long-lasting lethargy, myalgia, weakness, hopelessness, psychological distress, and sleep abnormalities which may overlay with the clinical and sleep topographies of fibromyalgia and chronic fatigue syndrome [25]. Myopathy due to corticosteroid use, muscle degenerative changes, and weakness has similarly been described in ARDS survivors during 1-year follow-up period [26]. Another study on the survivors of SARS confirmed deficits in cardio-respiratory performance in 6-min walking test, abnormalities in the musculo-skeletal performance, and quality of life impairment [27]. A similar image was described subsequently after the H1N1 influenza epidemic in 2009 [28]. Following SARS, some cases suffered a decline in their mental well-being during 1-year follow-up period comprising nervousness, hopelessness, high incidence of posttraumatic stress disorders, and psychosis [29].

Moreover, 10 years following SARS recovery, vulnerability to lung contagions, abnormality in glucose absorption, and elevated levels of phosphatidylinositol persist in comparison with healthy ones [30]. A recent meta-analysis found that 25% of SARS and MERS survivors had diminished lung function, quality of life, and exercise capability at 6 months post-discharge [31]. Similarly, the MERS convalescent cases also reported the ominously lower quality physical health for at least 14 months after the infection start, also survivors who anticipated intensive care unit admittance described an ominously minor inclusive quality of life than those with non-critical disease [32].

In the current study, 29.1% of included cases had breathing difficulty. This may be explained by some persistent fibrotic changes in the lungs of COVID-19 recovered patients following the current management and discharge rules which may disturb their respiratory function [33]. Moreover, patients with severe COVID-19 criteria may progress to acute respiratory distress syndrome (ARDS) and necessitate mechanical ventilation. ARDS may cause indefinite lung damage, contributing to persistent respiratory disorders after convalescence [34]. Amid 33 and 75% of cases with COVID-19 necessitate mechanical ventilation, often for a long period, and there are substantial long-term effects allied with prolonged period of intubation [35, 36]. Those on ventilators are more susceptible to respiratory contagions, which, consecutively, making patients more vulnerable to further risk of irreversible damage of the lung tissue.

There is emergent proof advocating that pulmonary thrombo-embolism is likely an underreported complication allied with COVID-19 that carries actually a major risk of long-standing pulmonary hypertension [37]. Furthermore, prior studies have shown that acute lung injury is allied with pulmonary fibrosis on CT scans and associates with restrictive functional pattern and worse quality of life [38].

Thirty-five percent of cases included in this study had nervousness and hopelessness. Consistently, COVID-19 is linked with a major mental health problem in both the acute stage and the chronic term [39]. Nervousness, hopelessness, post-traumatic stress disorder, and sleeplessness are common behind severe coronavirus contagions [40, 41]. Thirty percent of the initial 153 COVID-19 patients in the UK had psychological health troubles comprising neurosis, decline in cognitive functions, and other disorders [39]. Corticosteroid therapy is also associated with the progression of psychotic complaints [42]. Correspondingly, after SARS, 5–44% complained of several mental disorders at 1 year comprising nervousness, hopelessness, psychosis, and greater rates of post-trauma stress syndrome [29].

Myalgia (60.0%) and arthralgia (57.2%) were common complaints among the patients included in this study. Correspondingly, post-COVID-19 long-lasting pain may distress patients of various age groups, but the elderly patients are the most commonly affected [43]. Similarly, after the acute SARS, some patients may evolve a chronic fatigue syndrome/myalgic encephalomyelitis (CFS/ME)—like a disease with worse sleep quality, lethargy, myalgia, and hopelessness, with some incapable of coming back to their work [25].

Finally, the results of this study are challenged by some *limitations*. First, the designated sample of post-COVID-19 cases is not entirely illustrative of all post-COVID-19 patients. Second, symptoms that initiated after the date of analysis were not verified in this survey. Third, random selection bias may be present and an inability for personal face-to-face interviews in some cases. Finally, our results were made as a single point of follow-up, and further follow-up at 3, 6, or 12 months would aid further understanding of the progression of symptoms post-COVID-19. So, more studies and researches are desired to better appreciate, describe, and identify the persistent post-COVID symptoms in various sceneries and residents.

## Conclusions

COVID-19 is an emerging disease that can present with a diverse spectrum of long-term post-COVID-19 symptoms. All patients may experience post-COVID-19 symptoms; however, increased acute phase symptom severity and score above 18 together with the presence of any comorbid diseases increase the risk for persistent post-COVID-19 manifestations and severity. Physicians and health care employees should aware patients, especially the high-risk group, of the probable long-lasting problems of COVID-19 and reassure them to pursue medical care for any condition they may progress. Moreover, we recommend initiating multi-disciplinary post-COVID-19 recovery units’ emphases on prompt and regular communication and socialization, along with physical and neurological assessments and management together with increasing scope for further detailed researches throughout those units.

## Data Availability

The datasets used and/or analyzed during the current study are available from the corresponding author on reasonable request.

## Abbreviations

DM: Diabetes mellitus
PCR: Polymerase chain reaction
BMI: Body mass index
GIT: Gastrointestinal symptoms
ROC curve: Receiver operating characteristic curve
CFS/ME: Chronic fatigue syndrome/myalgic encephalomyelitis
SARS: Severe acute respiratory syndrome
MERS: Middle East respiratory syndrome
ARDS: Acute respiratory distress syndrome
ICU: Intensive care unit
PICS: Post-intensive care syndrome
